# Supplementary material for: Endosome rupture enables enteroviruses from the family Picornaviridae to infect cells
Source: Commun Biol. 2024 Nov 8;7:1465. doi: 10.1038/s42003-024-07147-9 (PMC11543853; doi:10.1038/s42003-024-07147-9)
Supplement: Supplementary file 2 — Supplementary Information [file 42003_2024_7147_MOESM2_ESM.pdf]

Supplementary Information for:

Endosome rupture enables enteroviruses from the family *Picornaviridae* to infect cells

Short title: Enterovirus cell entry visualized *in situ*

Aygul Ishemgulova[1&]*, Liya Mukhamedova[1&], Zuzana Trebichalská[1,2&], Veronika Rájecká[1], Pavel Payne[1], Lenka Šmerdová[1], Jana Moravcová[1], Dominik Hrebík[1], David Buchta[1], Karel Škubník[1], Tibor Füzik[1], Štěpánka Vaňáčová[1], Jiří Nováček[1], Pavel Plevka[1]*

[1] Central European Institute of Technology, Masaryk University, Kamenice 5, Brno, 625 00, Czech Republic
[2] National Centre for Biomolecular Research, Faculty of Science, Masaryk University, Kamenice 5, 625 00, Brno, Czech Republic
[&] These authors contributed equally
* Corresponding authors: aishemgulova@gmail.com, pavel.plevka@ceitec.muni.cz

Content:

Supplementary figures
Supplementary tables
Legends to supplementary movies

Supplementary figures

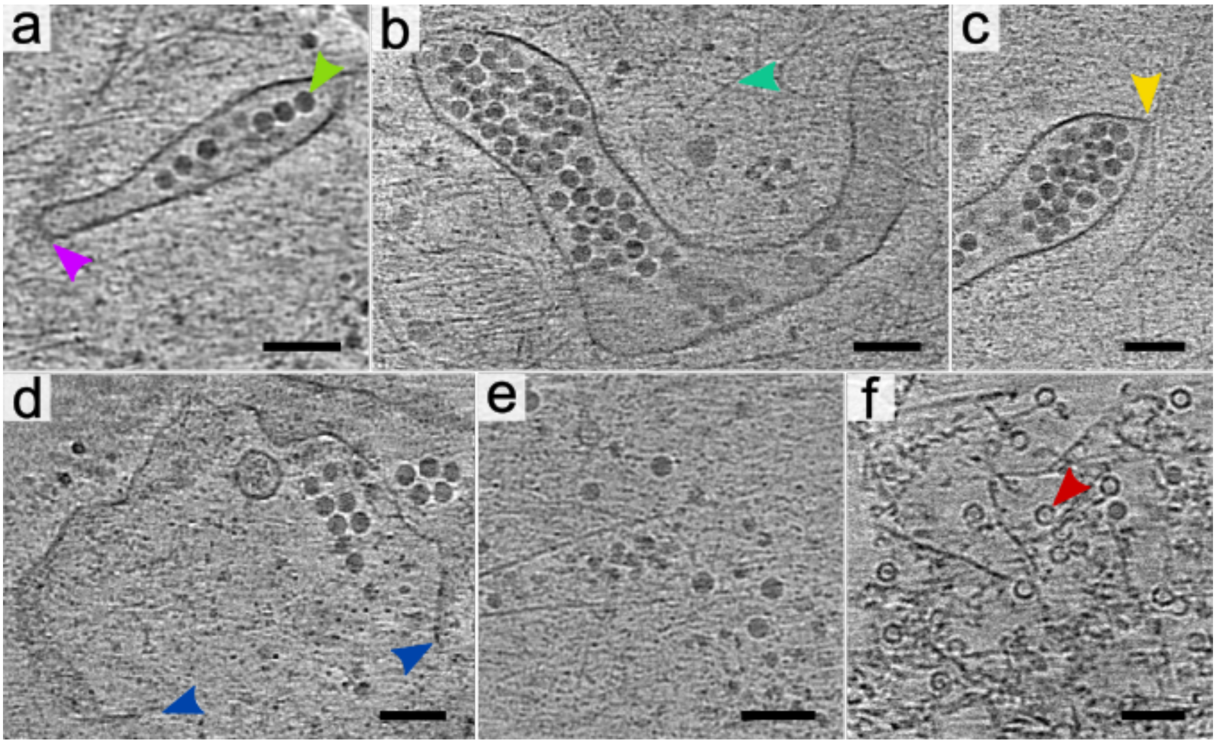

Supplementary Fig. 1. Release of echovirus 18 from endosomes into the cytoplasm. The images show 1.6 nm thick tomographic slices of echovirus 18-infected cos-7 cells 20-30 min post-infection. **(ab)** Endosomes containing echovirus 18 particles. The segment of the endosome covered with a protein coat in panel (a) is indicated by a magenta arrowhead. A selected virion is indicated by a green arrowhead. A selected actin microfilament in panel (b) is indicated by a cyan arrowhead. **(c)** Endosome with warped membrane forming a cone-shaped protrusion. The tip of the cone adjacent to the actin microfilament is indicated by a yellow arrowhead. **(d)** Ruptured endosome releasing echovirus 18 into the cytoplasm. The edges of the vesicle opening are indicated by blue arrowheads. **(ef)** Virions and empty particles of echovirus 18 in the cell cytoplasm. Scale bar 100 nm.

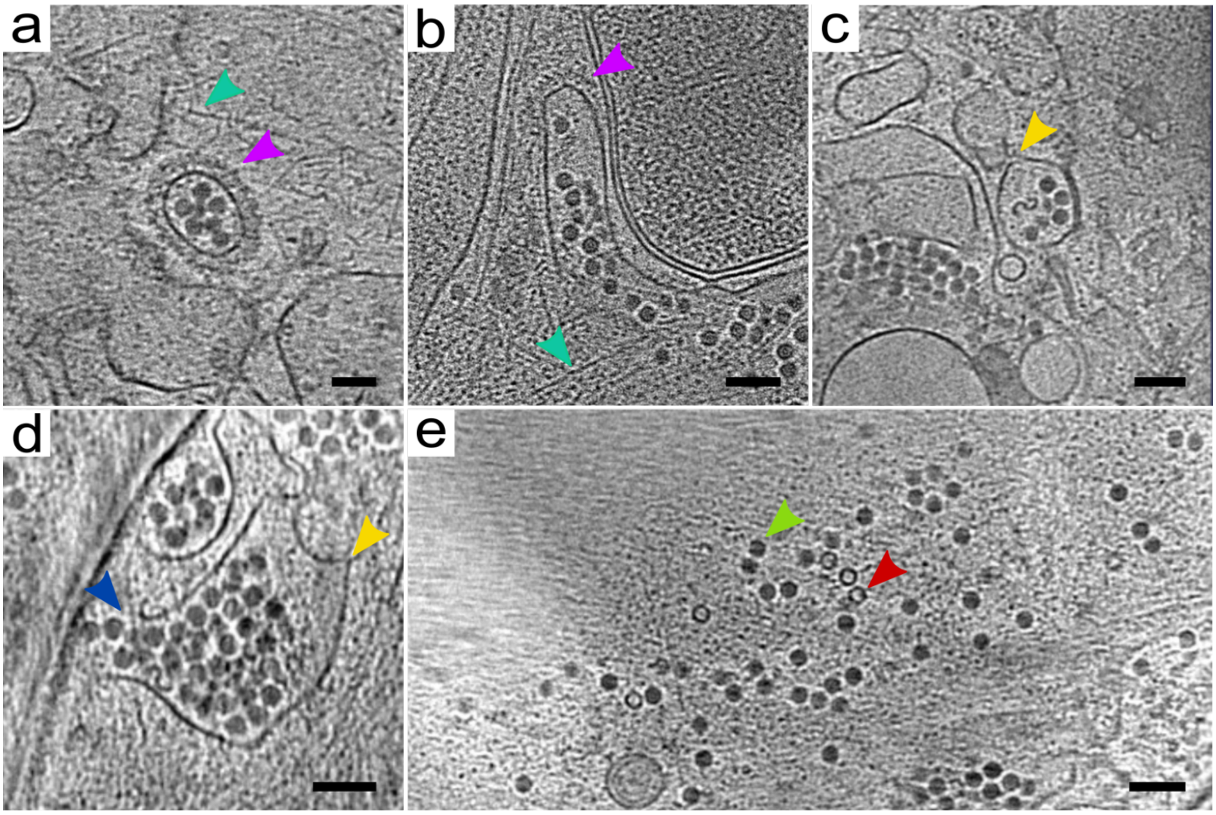

Supplementary Fig. 2. Release of echovirus 30 from endosomes into the cytoplasm. The images show 1.6-nm-thick tomographic slices of echovirus 30-infected cos-7 cells 30-60 min post-infection. **(a)** Endosome containing echovirus 30 particles. A segment of the endosome covered with a protein coat is indicated by magenta arrowhead. A selected actin microfilament is indicated by a cyan arrowhead. **(b)** Ruptured endosome with part of its membrane covered with a protein coat. The protein coat is indicated by a magenta arrowhead, a selected actin filament is indicated by a cyan arrowhead. **(c)** Endosome with warped membrane forming a cone-shaped protrusion. The tip of the cone is indicated by a yellow arrowhead. **(d)** Ruptured endosome releasing echovirus 30 into the cytoplasm. The opening in the endosome membrane is indicated by a blue arrowhead. **(e)** Virions and empty particles of echovirus 30 in the cell cytoplasm. Scale bar 100 nm.

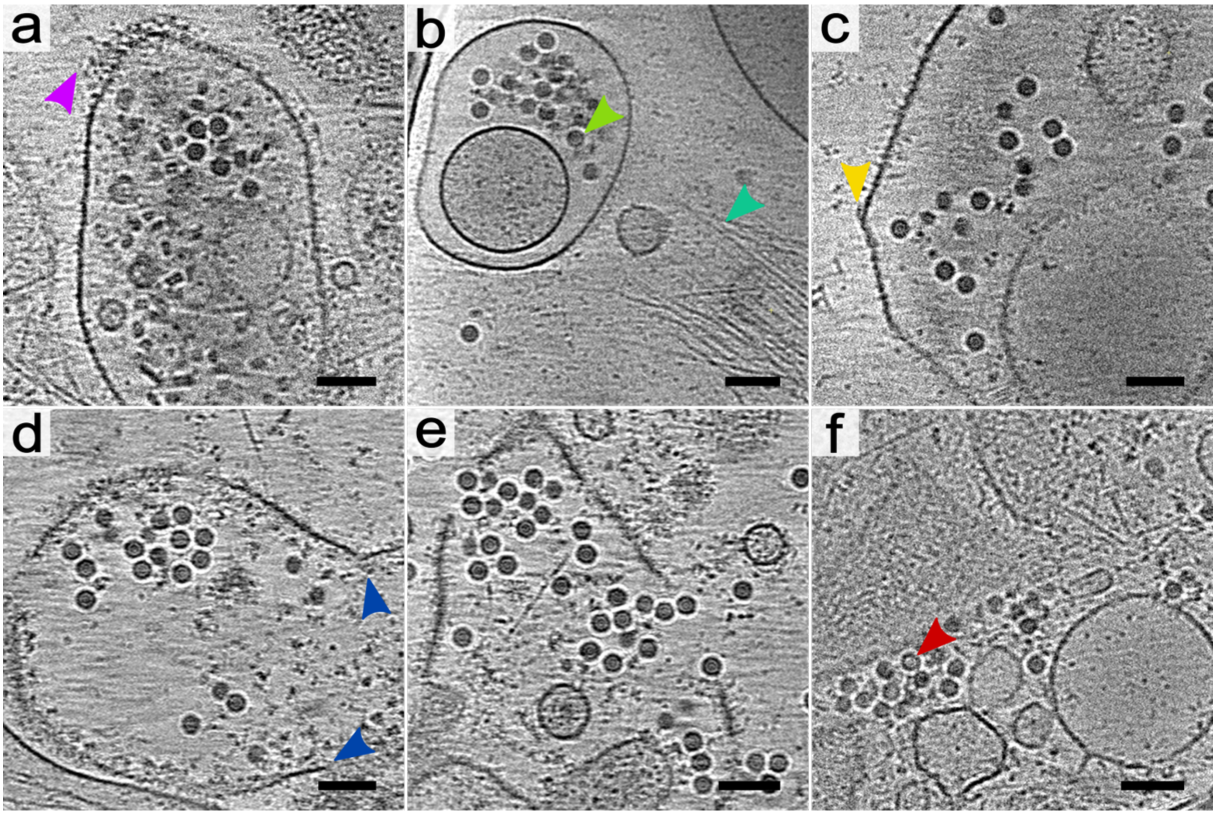

Supplementary Fig. 3. Release of enterovirus 71 from endosomes into the cytoplasm. The images show 1.6-nm-thick tomographic slices of enterovirus 71-infected cos-7 cells 30-60 min post-infection. **(a)** Endosome containing enterovirus 71 particles. A segment of the endosome covered with a protein coat is indicated by a magenta arrowhead. **(b)** Double-membrane endosome containing virus particles. Selected virion and actin microfilament are indicated by green and cyan arrowheads, respectively. **(c)** Endosome with warped membrane forming a cone-shaped protrusion. The tip of the cone is indicated by a yellow arrowhead. **(d)** Ruptured endosome releasing enterovirus 71 into the cytoplasm. The edges of the opening in the endosome membrane are indicated by blue arrowheads. **(ef)** Virions and empty particles in the cell cytoplasm. Selected empty particle in panel (f) is indicated by a red arrowhead. Scale bar 100 nm.

a

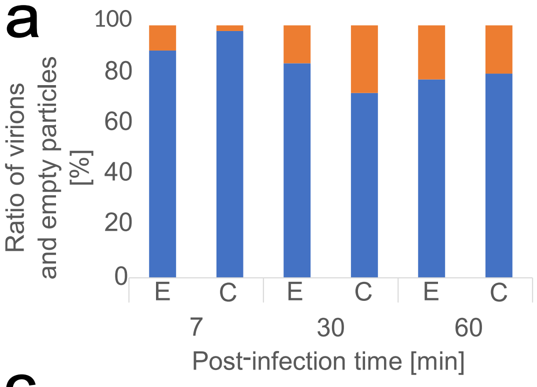

b

Human rhinovirus 2						
Post-infection time [min]	7		30		60	
Cellular compartment	Endosome	Cytoplasm	Endosome	Cytoplasm	Endosome	Cytoplasm
Empty particles	2 (0.8%)	2 (10.0%)	11 (2.2%)	42 (15.0%)	49 (26.8%)	77 (21.4%)
Virions	261 (99.2%)	18 (90.0%)	480 (97.8%)	238 (85.0%)	134 (73.2%)	283 (78.6%)

c

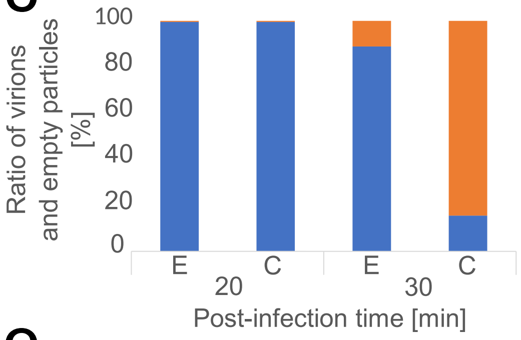

d

Echovirus 18				
Post-infection time [min]	20		30	
Cellular compartment	Endosome	Cytoplasm	Endosome	Cytoplasm
Empty particles	10 (0.5%)	20 (0.4%)	1 (11.1%)	4071 (84.5%)
Virions	2117 (99.5%)	4544 (99.6%)	8 (88.9%)	749 (15.5%)

e

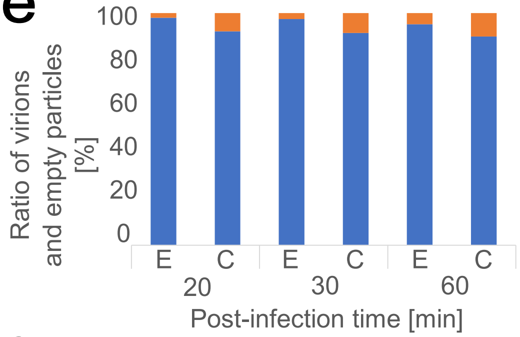

f

Echovirus 30						
Post-infection time [min]	20		30		60	
Cellular compartment	Endosome	Cytoplasm	Endosome	Cytoplasm	Endosome	Cytoplasm
Empty particles	11 (2.0%)	3 (7.9%)	18 (2.5%)	17 (8.6%)	43 (4.8%)	100 (10.1%)
Virions	534 (98.0%)	35 (92.1%)	688 (97.5%)	181 (91.4%)	857 (95.2%)	888 (89.9%)

g

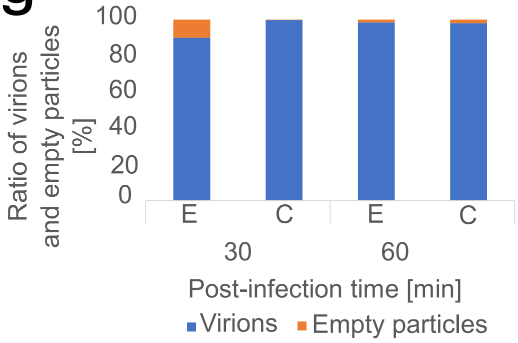

h

Enterovirus 71				
Post-infection time [min]	30		60	
Cellular compartment	Endosome	Cytoplasm	Endosome	Cytoplasm
Empty particles	3 (10.0%)	18 (0.3%)	4 (1.5%)	36 (2.0%)
Virions	27 (90.0%)	6748 (99.7%)	255 (98.5%)	1805 (98.0%)

Supplementary Fig. 4. Fractions of virions and empty particles in endosomes and cytoplasm of infected cells. The graphs show the fractions of virions (blue) and empty particles (orange) in the cytoplasm (C) and endosomes (E) of infected cos-7 cells. The localizations of virus particles were determined by the visual inspection of electron tomograms of infected cells. Particles with uncertain localizations (10%) were omitted from the analyses. The tables show absolute numbers of counted particles, the numbers in brackets indicate percentages of the particle types in each cellular compartment at a given time. Cells infected by **(ab)** rhinovirus 2, **(cd)** echovirus 18, **(ef)** echovirus 30, and **(gh)** enterovirus 71.

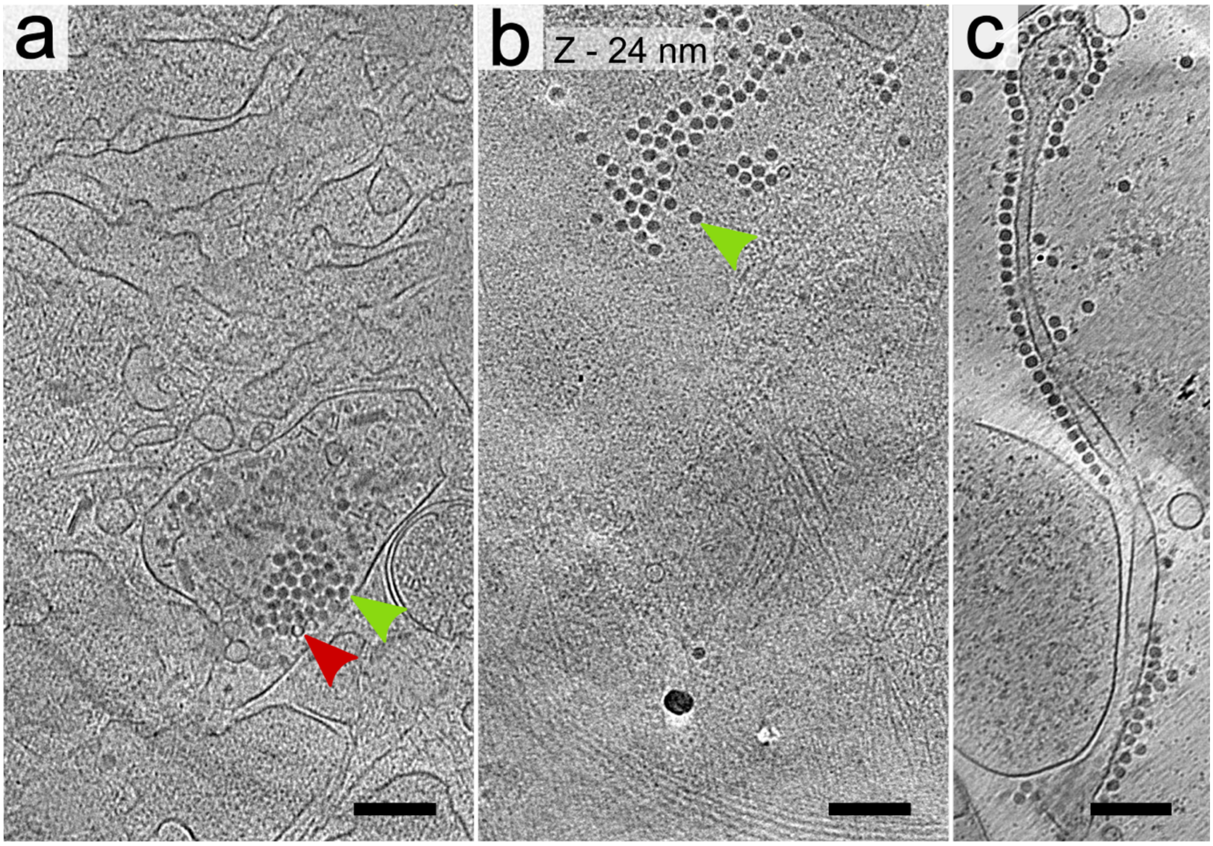

Supplementary Fig. 5. Attachment of enteroviruses to the cytoplasmatic membrane. The images show 1.6-nm-thick tomographic slices of cos-7 cells. **(ab)** Two tomographic slices of the same segment of echovirus 30-infected cos-7 cell showing (a) interior of the cell with virus particles in an endosome and (b) cell surface with attached virions. The green arrowheads indicate selected virions and the red arrowhead indicates a selected empty particle. **(c)** Filopodium of a cos-7 cell with virions of enterovirus 71 attached to its surface. Scale bar 200 nm.

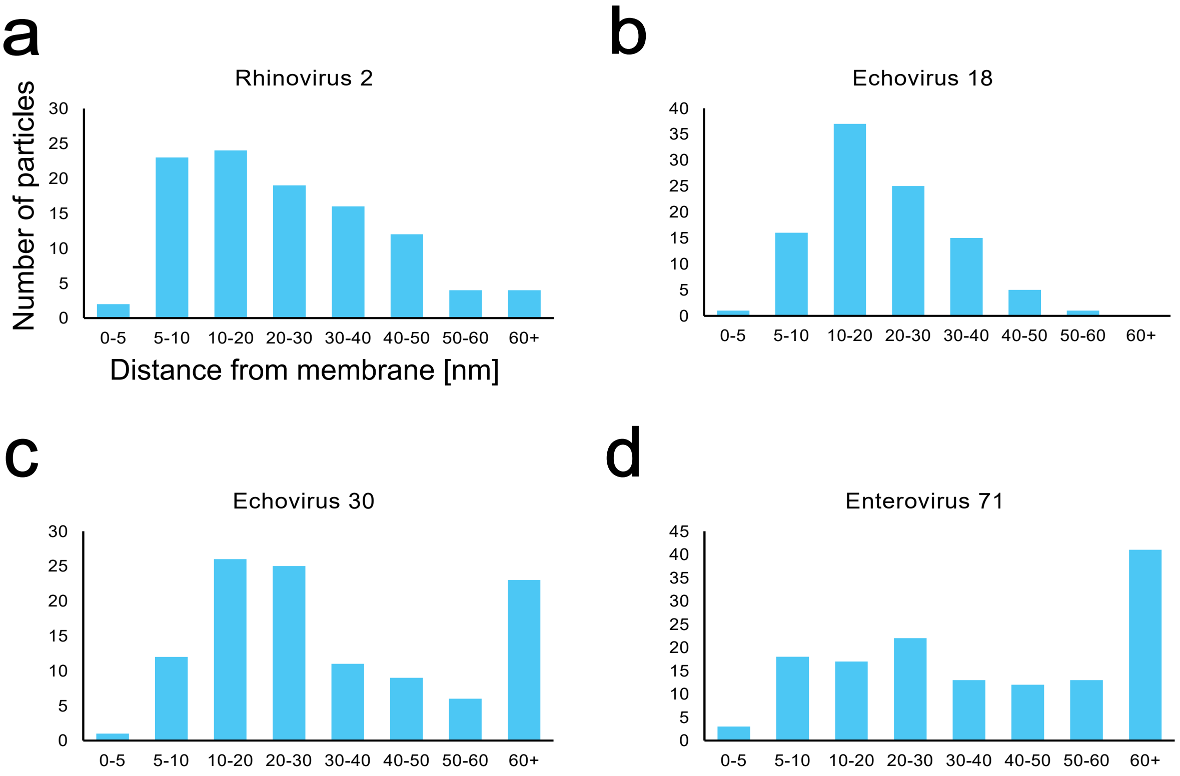

e

Virus	Distance from membrane [nm] (Mean ± SD)
Rhinovirus 2	33 ± 15
Echovirus 18	21 ± 11
Echovirus 30	37 ± 28
Enterovirus 71	54 ± 39

Supplementary Fig. 6. Distances of enterovirus particles in endosomes from membranes. (a-d) Histograms of the distributions of distances of virus particles from the closest endosome membrane: (a) rhinovirus 2, (b) echovirus 18, (c) echovirus 30, and (d) enterovirus 71. (e) The table shows the mean distances of enterovirus particles from the closest endosome membrane and standard deviations. The differences between the studied enteroviruses were not significant.

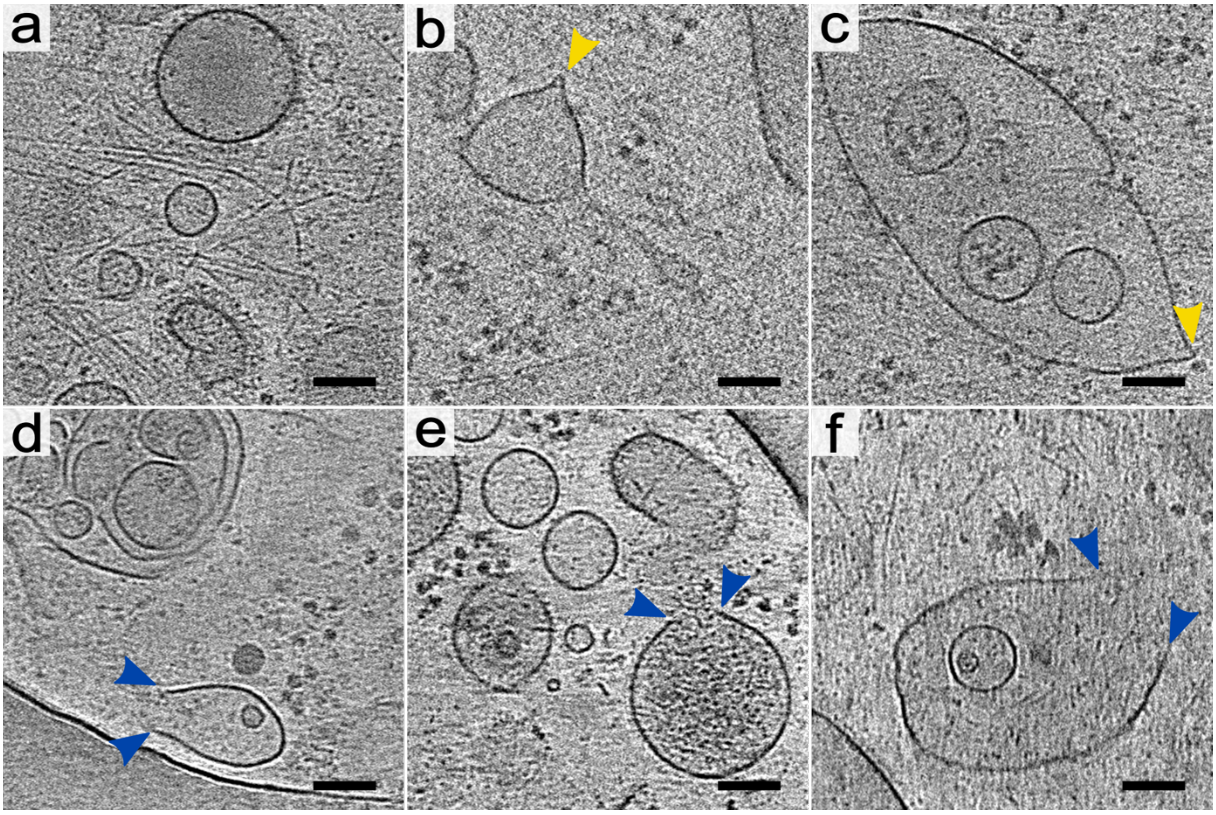

Supplementary Fig. 7. Warping and rupture of endosomes in control cells. The images show 1.6-nm-thick tomographic slices of cos-7 cells. **(a)** Endosomes with smooth membranes. **(bc)** Endosomes with warped membranes (tips of membrane cones are indicated by yellow arrowheads). **(d-f)** Disrupted endosomes (openings in the endosome membranes are indicated by blue arrowheads). Scale bar 100 nm.

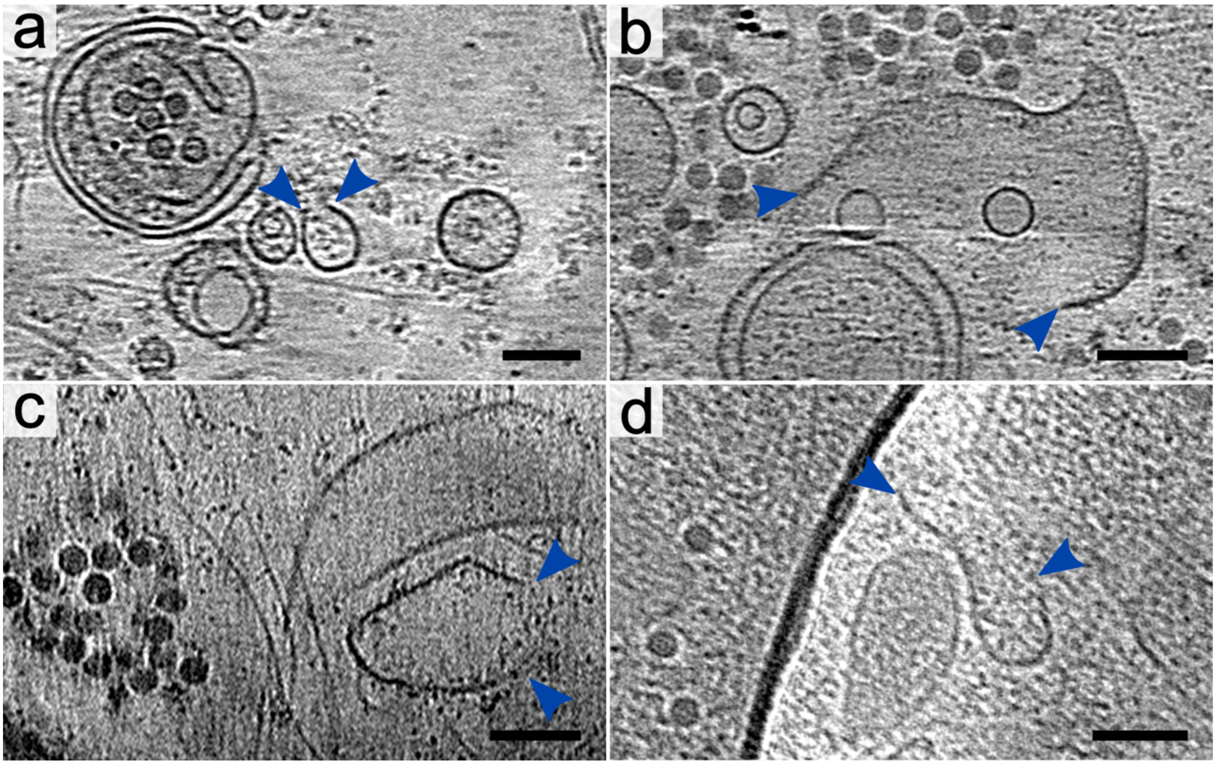

Supplementary Fig. 8. Infected cells contained disrupted endosomes with no virus particles inside. The images show 1.6-nm-thick sections from tomograms of cells infected by **(a)** rhinovirus 2, **(b)** echovirus 18, **(c)** enterovirus 71, and **(d)** echovirus 30. The edges of the ruptured endosome membranes are indicated by blue arrowheads. Scale bars 100 nm.

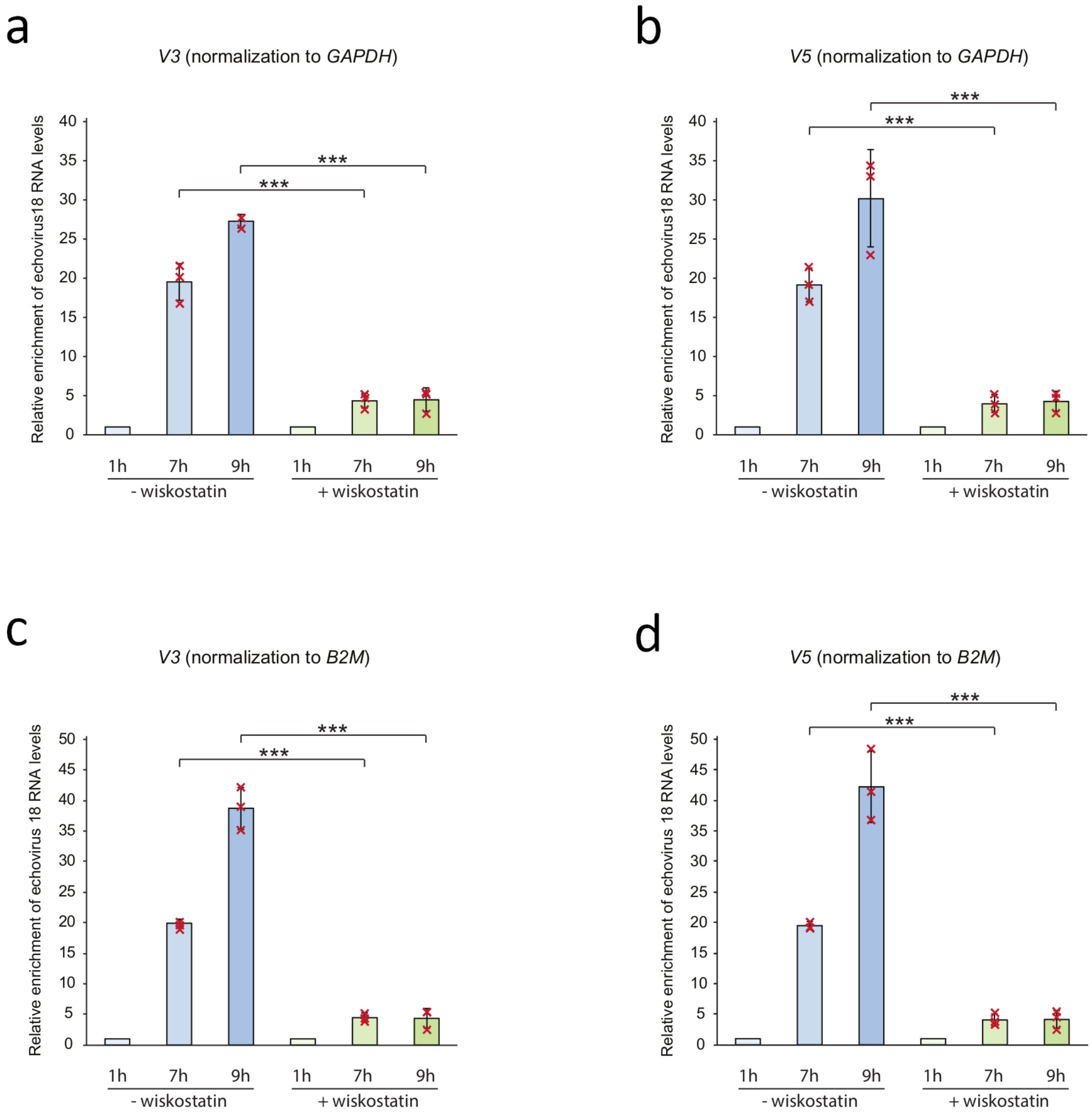

Supplementary Fig. 9. Wiskostatin inhibits infection of cos-7 cells by echovirus 18. RT-qPCR analysis of relative abundance of echovirus 18 RNA genomes in infected cos-7 cells using the standard Livak method. Relative levels of echovirus 18 genomes in control cells and cells treated with 50 µM wiskostatin 30 min before infection are shown at 1, 7, and 9 hours post-infection. The amounts of the virus genomes were quantified using V3 **(ac)** and V5 **(bd)** pairs of primers targeting distinct regions in echovirus 18 genome. The amounts of virus genomes were normalized by the ΔΔCt method to the house-keeping genes *glyceraldehyde-3-phosphate dehydrogenase* (GAPDH) (ab) or *β-2 microglobulin* (B2M) (cd) and to the time point of 1-hour post-infection. Error bars represent standard deviations for three independent biological replicates. *** indicates p<0,005. p-values of the individual comparisons are provided in supplementary material to the article. Primer sequences are listed in Table S3. Please see Materials and methods for details.

dCt (normalized to GAPDH or B2M)

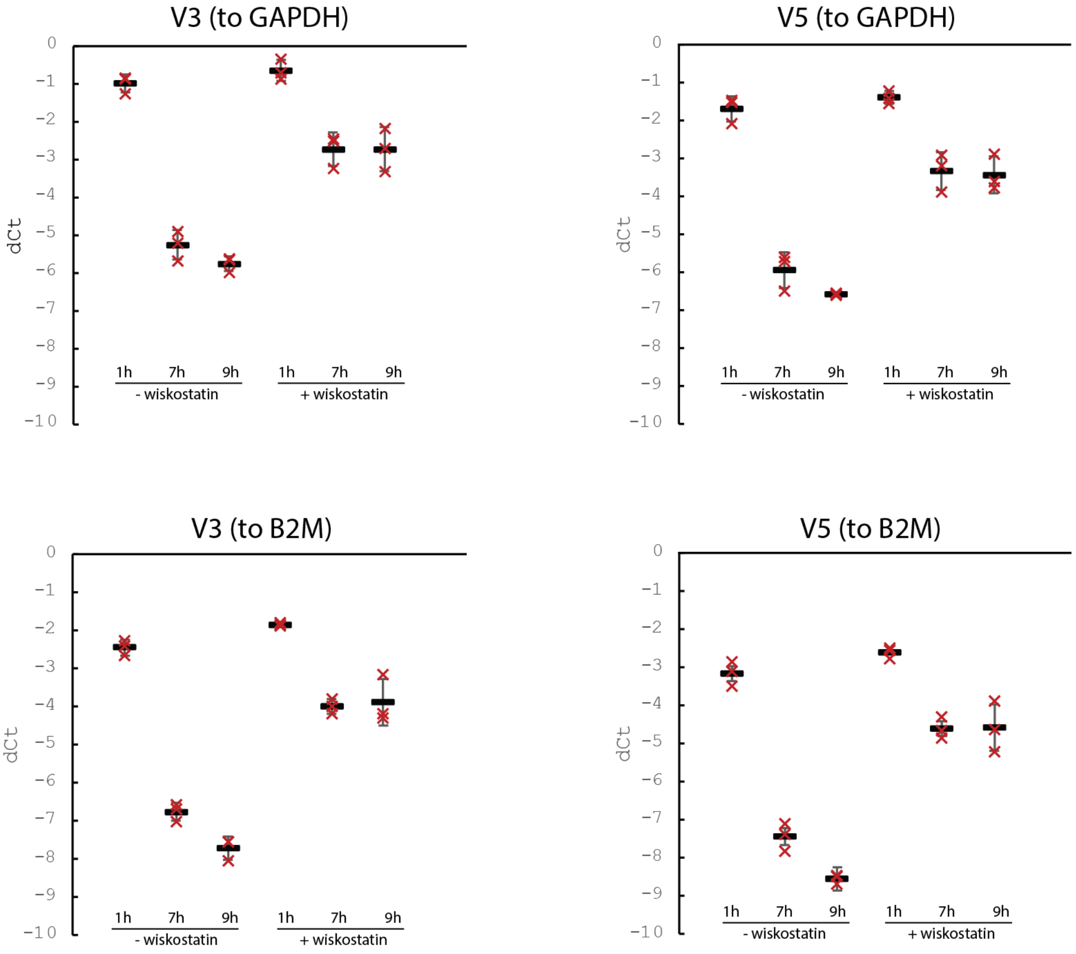

Supplementary Fig. 10. Source data for determining the wiskostatin inhibition effect on infection of cos-7 cells by echovirus 18 presented in Supplementary Fig. S9.

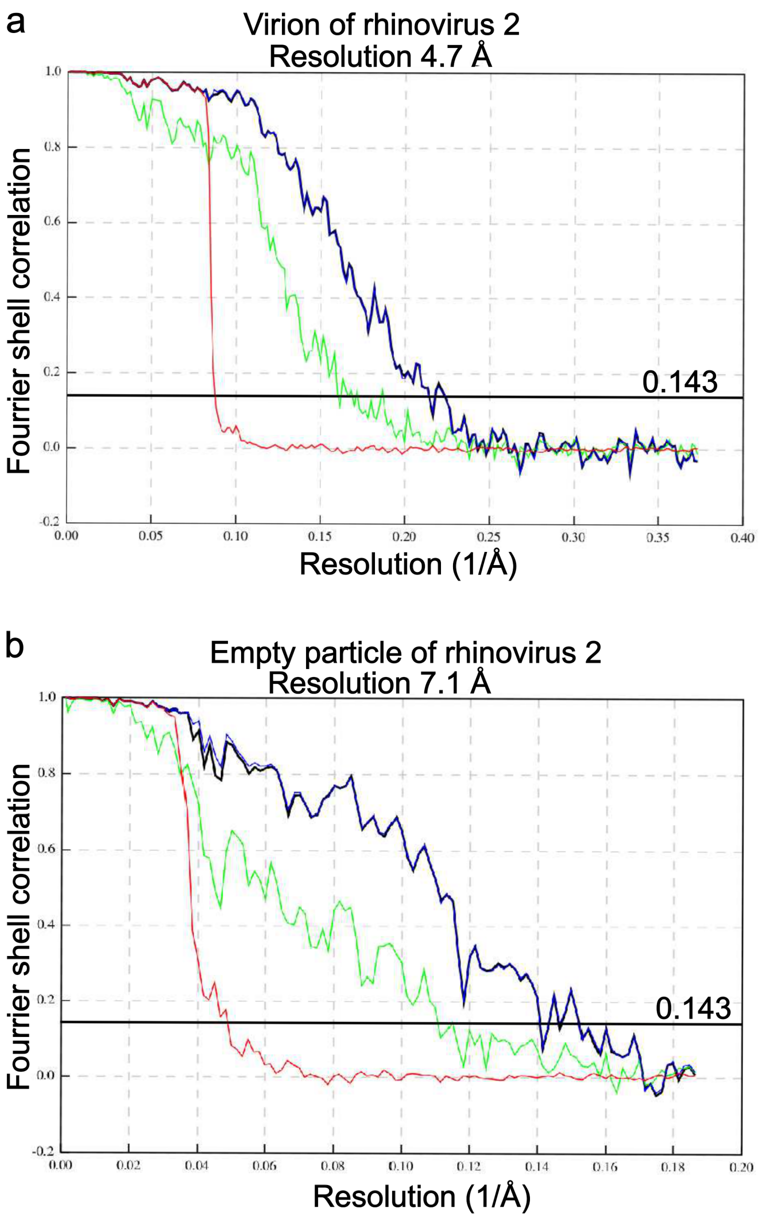

Supplementary Fig. 11. FSC curves of virion (a) and empty particle (b) of rhinovirus 2 reconstructed using micrographs recorded on infected cos-7 cells. Fourier shell correlation curves of Fourier shell correlation corrected half-maps (black), unmasked half-maps (green), masked half-maps (blue), and phase-randomized-masked half-maps (red) of individual cryo-EM reconstructions. The horizontal black line indicates the reference FSC value of 0.143. The final resolution is reported for the FSC cutoff at 0.143.

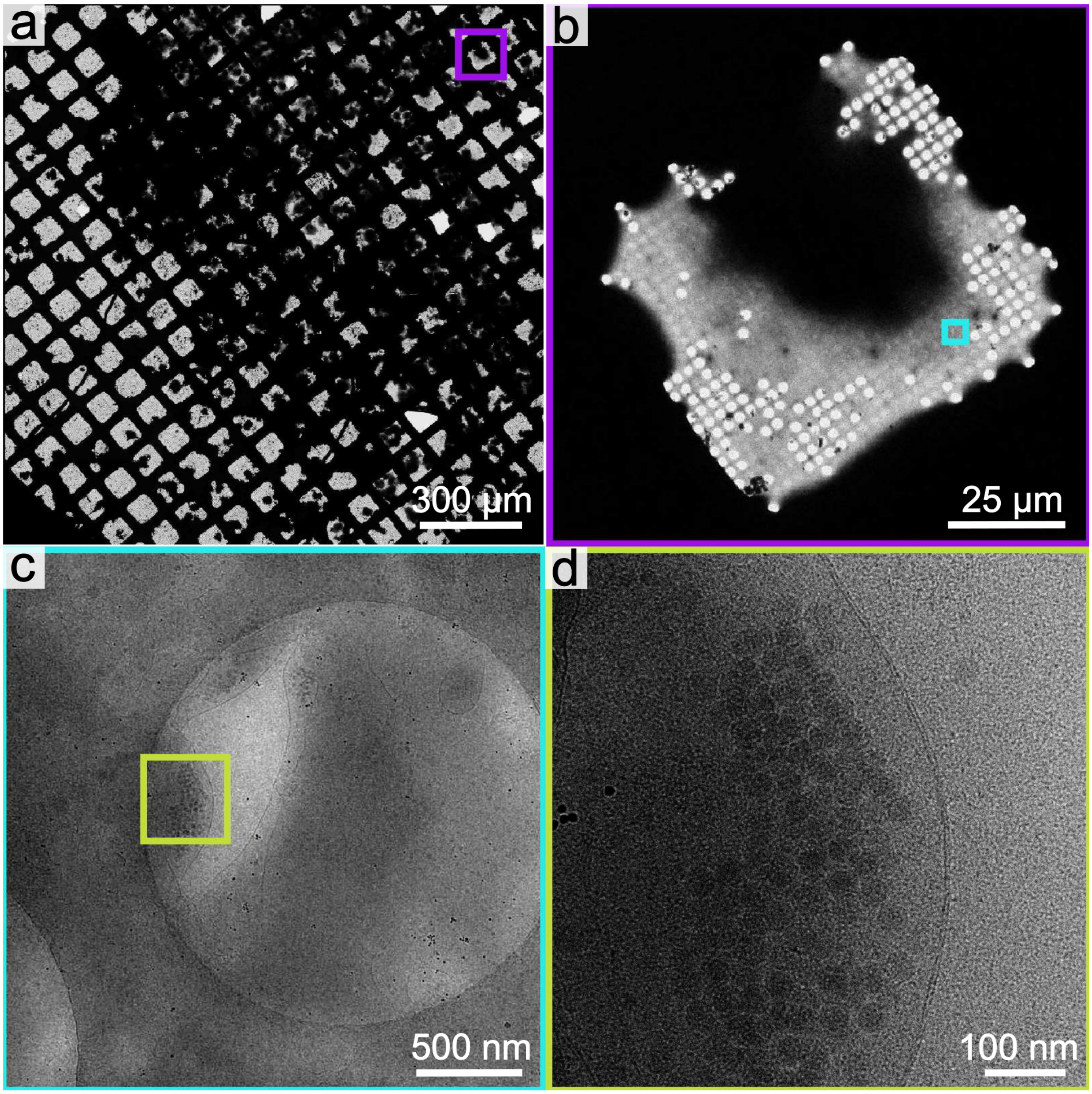

Supplementary Fig. 12. Series of images demonstrating the process of selection of an area for collecting tomographic tilt series of enterovirus-infected cos-7 cells. Panels (a-d) show a series of electron micrographs of cos-7 cells vitrified on a grid for electron microscopy recorded with increasing magnification. Every subsequent panel shows a magnified region indicated by a colored square in the previous one. **(a)** Overview of electron microscopy grid with cos-7 cells. **(b)** Image of a grid square with cos-7 cell. The dark area in the middle of the square represents a thick and electron non-transparent region of the cell. Grey areas represent cellular lamellipodia that are thin and electron-transparent. **(c)** Detail of cell lamellipodium with particles of rhinovirus 2 in vesicles. **(d)** High-magnification image of a segment of a vesicle containing rhinovirus 2.

Supplementary tables

Supplementary Table 1. Comparison of relative abundances of smooth, warped, and ruptured endosomes in infected and control cells.

Virus	Post-infection time [min]	Empty endosomes			Virus-containing endosomes		
		Smooth	Warping	Ruptured	Smooth	Warping	Ruptured
Human rhinovirus 2	7	60 % (6)	40 % (4)	0 % (0)	14 % (1)	86 % (6)	0 % (0)
	30	72 % (56)	23 % (18)	5 % (4)	56 % (10)	39 % (7)	5 % (1)
	60	63 % (397)	31 % (199)	6 % (38)	49 % (53)	29 % (32)	22 % (24)
Echovirus 18	30	71 % (175)	24 % (59)	5 % (12)	45 % (9)	45 % (9)	10 % (2)
	60	78 % (36)	11 % (5)	11 % (5)	75 % (3)	25 % (1)	0 % (0)
Echovirus 30	20	70 % (40)	26 % (15)	4 % (2)	50 % (3)	50 % (3)	0 % (0)
	30	56 % (43)	34 % (26)	10 % (8)	0 % (0)	60 % (3)	40 % (2)
	60	69 % (157)	21 % (49)	10 % (23)	8 % (1)	25 % (3)	67 % (8)
Enteroviru s 71	30	57 % (158)	39 % (108)	4 % (12)	42 % (5)	42 % (5)	16 % (2)
	60	68 % (76)	29 % (33)	3 % (3)	33 % (3)	56 % (5)	11 % (1)
Non-infected		65 % (381)	23 % (138)	12 % (69)			

For infected cells, the relative abundances are reported separately for empty endosomes and endosomes containing virus particles. The numbers in brackets indicate the absolute numbers of endosomes counted.

Supplementary Table 2. Particle counts, and the numbers of tomograms used to determine the effects of wiskostatin and bafilomycin on the probability of genome release from individual particles.

	Virions in cytoplasm	Empty particles in cytoplasm	Virions in vesicles	Empty particles in vesicles	Total particles	Number of analyzed tomograms
Infected untreated cells	233	34	659	156	1082	23
Infected wiskostatin-treated cells	98	142	362	812	1414	16
Infected bafilomycin A1-treated cells	453	31	402	6	892	11

Supplementary Table 3. Primers used for RT-qPCR quantification of the effect of wiskostatin on enterovirus infection.

Target gene	Primer name	Forward primer sequence	Reverse primer sequence	Product length, bp
echovirus 18	V3	CCGGTCAATTGGCTACCTTA	TGTCCATGCACTCTTTCAGC	195
echovirus 18	V5	CACACCATTCATGGCTCAAC	ACTGGGATCCACTTGTGAGG	151
GAPDH	GAPDH	GGACCTGACCTGCCGTC	GGAAGAGTGGGTGTCGCTG	152
β-2 microglobulin	B2M	CGTGCTCCAAAGATTCAGGT	ACGGCAGGCATACTCATCTT	237

Supplementary Table 4. Cross-correlation coefficients comparing *in situ* cryo-EM reconstruction of genome-containing particle to maps calculated from structures of rhinovirus 2 virion, activated particle, and empty particle determined by X-ray crystallography.

	X-ray structures:		
	Virion	Activated particle	Empty particle
Cryo-EM reconstruction of genome-containing particle	0.815	0.572	0.598
X-ray structures:			
Virion		0.689	
Activated particle			0.873

The cryo-EM reconstruction of the genome-containing particle was compared to maps calculated from PDB structures of the rhinovirus 2 virion (PDB code: 1FPN), activated particle (PDB code: 4L3B), and empty particle (PDB code: 3TN9) (*26, 30, 98*). Comparison of the maps calculated from PBD structures was used to determine the expected cross-correlation coefficients for capsids with similar structures (empty particle versus activated particle) and different structures (virion versus activated particle). For details, please see Supplementary materials and methods.

Supplementary Table 5. Cryo-EM data and structure quality indicators.

	Rhinovirus 2 virion (EMDB-15710) (PDB 8AY4)	Rhinovirus 2 empty particle (EMDB-15711) (PDB 8AY5)
**Data collection and processing**		
Magnification	105,000	105,000
Voltage (kV)	300	300
Electron exposure (e–/Å^2)	50	50
Defocus range (μm)	-1.8 to -3.0	-1.8 to -3.0
Pixel size (Å)	1.324	1.324
Symmetry imposed	icosahedral	icosahedral
Initial particle images (no.)	2,035	2,035
Final particle images (no.)	1,424	332
Map resolution (Å)	4.7	7.1
FSC threshold = 0.143		
Map resolution range (Å)	4.7-inf.	7.1-inf.
**Refinement**		
Initial model used (PDB code)	*de novo*	*de novo*
Model resolution (Å)	5.0	30.5
FSC threshold	0.5	0.5
Model resolution range (Å)	4.7-inf.	7.1-inf.
Map sharpening *B* factor (Å^2)	-143.27	-128.78
Model composition		
Non-hydrogen atoms	6143	5823
Protein residues	781	738
Ligands	NA	NA
B factors (Å^2)		
Protein	171.12	168.27
Ligand	NA	NA
R.m.s. deviations		
Bond lengths (Å)	0.012	0.012
Bond angles (°)	1.955	1.963
Validation		
MolProbity score	2.05 (100th percentile)	2.07 (100th percentile)
Clashscore	3.38 (100th percentile)	3.48 (100th percentile)
Poor rotamers (%)	3.08	3.25
Ramachandran plot		
Favored (%)	90.04	90.16
Allowed (%)	9.31	9.16
Disallowed (%)	0.65	0.68

Supplementary movie legends

Supplementary Movie 1: Cytoplasm of rhinovirus 2-infected cell imaged using cryo-ET. The movie shows a tomographic reconstruction of an infected cos-7 cell 60 min post-infection. The positions of virions, empty particles, ribosomes, and microfilaments were identified using template matching as implemented in emClarity (*121*), and the corresponding high-resolution structures were positioned into the tomogram. Microfilaments and membranes were segmented manually.

Supplementary Movie 2. Attachment of echovirus 30 to cytoplasmatic membrane. The movie shows tomographic slices of infected cos-7 cell 30 min post-infection. Virions of echovirus 30 are attached to the cell surface, whereas other particles are in an endosome inside the cell.
