# Supplementary material for: Endosome rupture enables enteroviruses from the family Picornaviridae to infect cells
Source: Commun Biol. 2024 Nov 8;7:1465. doi: 10.1038/s42003-024-07147-9 (PMC11543853; doi:10.1038/s42003-024-07147-9)
Supplement: Supplementary file 3 — Description of Additional Supplementary Files [file 42003_2024_7147_MOESM3_ESM.pdf]

Description of Additional Supplementary Files

File name: Supplementary Movie 1
Description: The movie shows a tomographic reconstruction of an infected cos-7 cell 60 min post-infection. The positions of virions, empty particles, ribosomes, and microfilaments were identified using template matching as implemented in emClarity (121), and the corresponding high-resolution structures were positioned into the tomogram. Microfilaments and membranes were segmented manually.

File name: Supplementary Movie 2
Description: The movie shows tomographic slices of infected cos-7 cell 30 min post-infection. Virions of echovirus 30 are attached to the cell surface, whereas other particles are in an endosome inside the cell.

File name: Supplementary Data 1
Description: Source data for plots and statistics presented in the paper
